# Prediagnosis Prostate-Specific Antigen Testing History in Patients With Incident Prostate Cancer

**DOI:** 10.1001/jamanetworkopen.2025.41321

**Published:** 2025-11-04

**Authors:** Lydia Guittet, Pierre Denis, Philippe Tuppin, Gaëlle Fiard, Romain Mathieu, Frédéric De Bels, Christine Le Bihan, Antoine Rachas

**Affiliations:** 1 French Public Health Insurance Fund (CNAM)/Strategy, Research and Statistics Departement (DSES), Paris, France; 2 Unité de Recherche Interdisciplinaire Pour la Prévention et le Traitement des Cancers (INSERM UMR 1086 ANTICIPE), Université Caen Normandie, Direction Recherche et Innovation du CHU Caen Normandie, Caen, France; 3 Recherche Translationnelle et Innovation en Médecine et Complexité (CNRS UMR 5525 TIMC), Team Gestes Médico-Chirurgicaux Assistés par Ordinateur (GMAO), Université Grenoble, Service de Chirurgie de l’urologie et de la transplantation rénale du CHU Grenoble-Alpes, Grenoble, France; 4 Institut de Recherche en Santé, Environnement et Travail (INSERM UMR 1085 IRSET), Team Epidémiologie et Science de L’exposition en Santé-Environnement (ELIXIR), Université Rennes, Service d’Urologie du Centre Hospitalier de Rennes, Rennes, France; 5 French National Cancer Institute (INCa), Boulogne-Billancourt, France

## Abstract

This cohort study evaluates prostate-specific antigen (PSA) testing history in patients with incident prostate cancer.

## Introduction

Although organized prostate-specific antigen (PSA) screening for prostate cancer (PCa) is not recommended by most health authorities, including those in France, a high frequency of PSA testing is observed in many countries with significant association with incidence but not PCa-specific mortality.^1,2,3^ However, ecological analysis in the US showed that the incidence of metastatic PCa, which had decreased during the first phase of intense PSA screening, has slightly increased since 2012.^4^

Little is known of the association between opportunistic PSA screening and incidence of late-stage PCa at the individual level. This study aimed to describe the trend in PSA testing history according to PCa stage at diagnosis.

## Methods

In this population-based nationwide cohort study based on the French National Health Data System (SNDS),^5^ data relating to health care reimbursements or registration for long-term disease over the period from 2011 to 2022 in men older than 40 years were analyzed to identify 2016 to 2022 incident PCa with no other cancer, using a 5-year washout period to exclude history of PCa. Since clinical stage was not reported in SNDS, the stage at diagnosis (localized, late-stage PCa [advanced or metastatic PCa]) was established based on the first-line treatment (eAppendix in Supplement 1). This report follows the Strengthening the Reporting of Observational Studies in Epidemiology (STROBE) reporting guideline. Caisse Nationale d’Assurance Maladie has permanent access to the SNDS by decree No. 2016-1871, so no patient consent or specific ethics committee approval were needed for this study.

Outpatient PSA test reimbursements were recorded for the 5 years preceding PCa diagnosis, excluding tests from the year immediately prior, as they may be directly related to the diagnosis. A history of 5 to 2 years of PSA testing was also recorded in men without PCa during the same period.

The average annual percentage changes (AAPC) for the age-adjusted proportions of 2- to 5-year prediagnosis PSA testing (≥1 test) stratified on stage at diagnosis were derived from logistic regression models adjusted on 5-year age groups. *t* Tests were used, and a 2-sided *P* < .05 was considered statistically significant. Data were analyzed in SAS Enterprise Guide version 8.6 (SAS Institute) from July 2024 to July 2025.

## Results

Among the 359 741 individuals with incident PCa diagnosis with a median (IQR) age of 70 (64-76) years, 240 605 (66.9%) had localized disease at diagnosis, 103 859 (28.9%) had late-stage disease, and 15 577 (5.3%) had disease that remained unstaged. Localized PCa was detected at a younger age than late-stage PCa (Figure).

**Figure.**
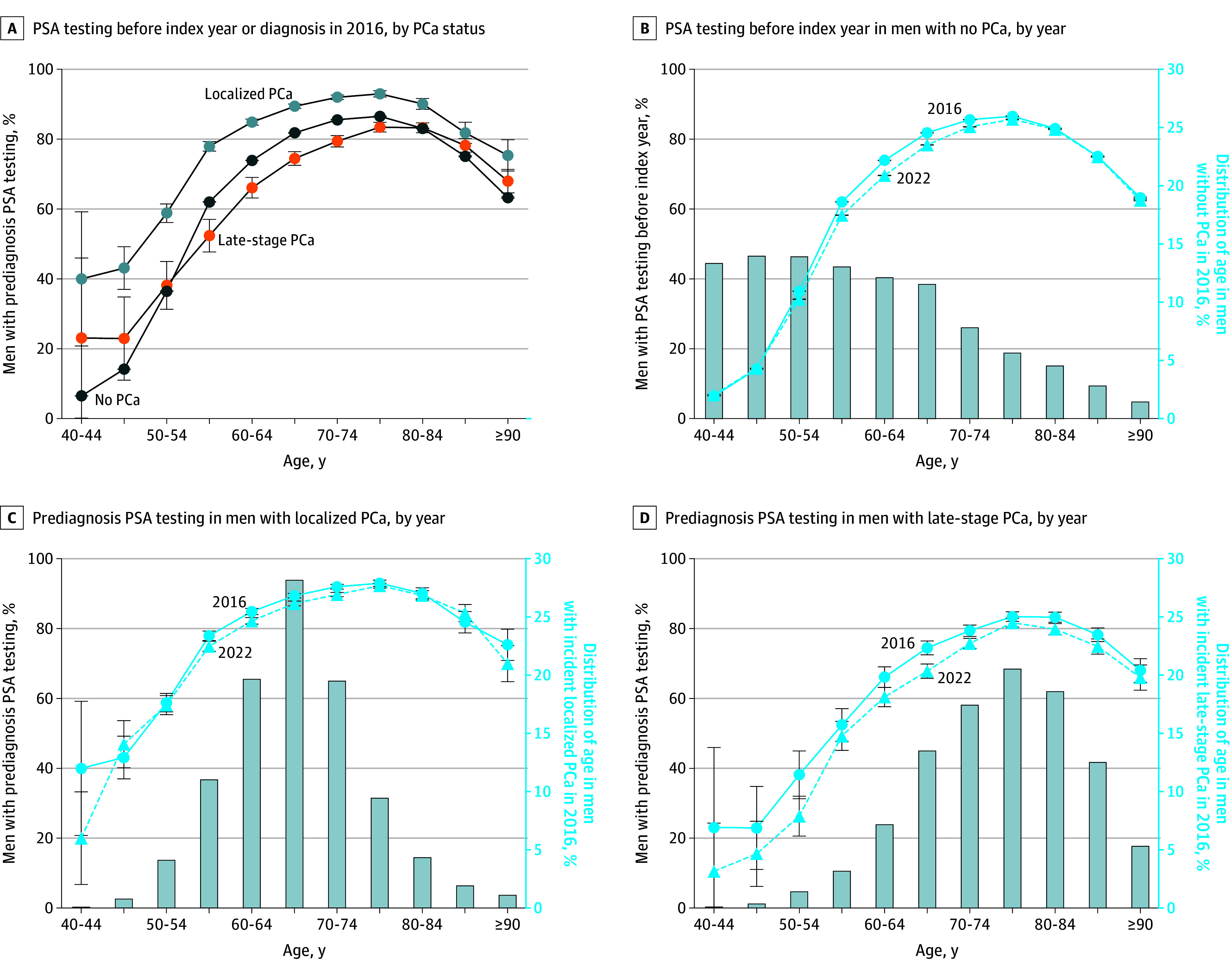
Prostate-Specific Antigen (PSA) Testing Before Index Year or Diagnosis Relative to Prostate Cancer (PCa), Age, and Year of Incidence A, Proportion of men who had 2 to 5 years prediagnosis PSA testing (or before index year in men with no PCa), stratified by PCa status and stage at diagnosis. B, Proportion of men with no PCa who had 2 to 5 years PSA testing before index year, stratified by year (lines). C, Proportion of men with incident localized PCa who had 2 to 5 years prediagnosis PSA testing stratified by year of incidence (lines), and distribution of age in 2016 (bars). D, Proportion of men with incident late-stage PCa who had 2 to 5 years prediagnosis PSA testing stratified by year of incidence (lines), and distribution of age in 2016 (bars). Error bars indicate 95% CIs.

In 2016, 27 949 (86.2%) individuals with localized PCa and 10 645 (76.9%) with late-stage PCa had 2- to 5-year prediagnosis PSA testing (Table). An increasing rate of 2- to 5-year PSA testing was observed with age until 80 years, with higher rates in men with localized PCa, then no PCa, and finally late-stage PCa (Figure). Overall, 2016 to 2022 AAPC for 2- to 5-year prediagnosis PSA testing was −0.29% (95% CI, −0.31% to −0.26%) for localized PCa and −0.67% (95% CI, −0.73% to −0.60%) for late-stage PCa (Table).

**Table.** Evolution of 2- to 5-Year Prediagnosis Prostate-Specific Antigen (PSA) Testing According to Age Group and Stage at Diagnosis, Compared With Men Without Prostate Cancer (PCa)

Status and age, y	Year	Men, No.	2- to 5-y Prediagnosis PSA testing (or before index year in men without PCa)		Penultimate year PSA testing before diagnosis (or before index year in men without PCa)	
			No. (%)	2016-2022 AAPC, % (95% CI)^a^	No. (%)	2016-2022 AAPC, % (95% CI)^b^
No prostate cancer						
≥40	2016	15 131 628	7 969 787 (52.7)	NA	4 541 196 (30.0)	−0.65 (−0.67 to −0.64)
	2022	16 437 818	8 555 867 (52.0)		4 830 675 (29.4)	
40-49	2016	4 126 296	429 723 (10.4)	NA	174 493 (4.2)	0.41 (0.41 to 0.41)
	2022	4 232 260	448 570 (10.6)		175 566 (4.2)	
50-74	2016	8 829 351	5 779 798 (65.5)	NA	3 305 027 (37.4)	−1.07 (−1.11 to −1.03)
	2022	9 660 366	6 060 260 (62.7)		3 384 485 (35.0)	
≥75	2016	2 175 981	1 760 266 (80.9)	NA	1 061 676 (48.8)	0.43 (0.42 to 0.43)
	2022	2 545 192	2 047 037 (80.4)		1 270 624 (49.9)	
Localized prostate cancer						
≥40	2016	32 435	27 949 (86.2)	−0.29 (−0.31 to −0.26)	20 091 (61.9)	−0.26 (−0.27 to −0.25)
	2022	38 353	32 706 (85.3)		23 952 (62.5)	
40-49	2016	218	119 (42.8)	−0.84 (−0.87 to −0.81)	71 (25.5)	−0.35 (−0.36 to −0.35)
	2022	246	106 (43.1)		67 (27.2)	
50-74	2016	26 722	22 949 (85.9)	−0.36 (−0.40 to −0.33)	16 401 (61.4)	−0.33 (−0.34 to −0.32)
	2022	29 559	24 901 (84.2)		18 095 (61.2)	
≥75	2016	5435	4881 (89.8)	0.04 (0.03 to 0.04)	3619 (66.6)	0.02 (0.02 to 0.02)
	2022	8548	7699 (90.1)		5790 (67.7)	
Late-stage prostate cancer^c^						
≥40	2016	13 845	10 645 (76.9)	−0.67 (−0.73 to −0.60)	7049 (50.9)	−0.76 (−0.79 to −0.73)
	2022	15 665	11 452 (73.1)		7519 (48.0)	
40-74	2016	5965	4279 (71.7)	−0.92 (−1.01 to −0.82)	2729 (45.8)	−0.93 (−0.97 to −0.90)
	2022	7433	4982 (67.0)		3171 (42.7)	
≥75	2016	7880	6366 (80.8)	−0.48 (−0.52 to −0.44)	4320 (54.8)	−0.64 (−0.67 to −0.62)
	2022	8232	6470 (78.6)		4348 (52.8)	

Abbreviations: AAPC, annual average percentage change; NA, not applicable.

^a^ AAPC in age-standardized proportions of men with 2 to 5 years of prediagnosis PSA testing history (5-year age distribution of PCa of relevant stage over the whole period as reference) and 95% CI derived from logistic regressions adjusted on 5-year age. Due to nonindependence of data in men without prostate cancer regarding 2- to 5-year history of PSA testing, who can be included in the analysis of several years, no logistic regression and no statistical tests were conducted for this population.

^b^ AAPC in age-standardized proportions of men with penultimate history of PSA testing history (5-year age distribution of relevant group over the whole period as reference) and 95% CI derived from logistic regressions adjusted on 5-year age.

^c^ Due to small number of incident late-stage prostate cancers in men aged 40 to 49 years (61 cases in 2016 and 77 in 2022), classes aged 40 to 49 and 50 to 74 years were grouped.

## Discussion

In this retrospective cohort study, a large proportion of men with PCa had undergone PSA testing during the 5 years before diagnosis, including those with late-stage PCa at diagnosis. Limited decreasing trends in PSA testing history were observed during the study period. This study provides nationwide epidemiological data for PCa over a 7-year period using exhaustive information on reimbursed health care delivery at individual level in the SNDS.

Limitations were common to studies based on claim databases and included the use of algorithms to record incident PCa and define stage at diagnosis, the lack of information on PSA test indication (sometimes unrelated to PCa screening^6^), and PSA results. Also, health care consumption outside of France and ethnicity remained unknown.

The higher proportion of localized PCa with 2- to 5-year prediagnosis PSA testing was expected, as they are mostly detected by screening, some as a result of overdiagnosis. Regarding late-stage PCa, this study contradicts the hypothesis that the majority of these cancers arise from missed opportunities for PSA screening, and suggests that rapidly progressing PCa is difficult to detect within the window of curability. PSA testing practices should be better standardized and accompanied by an organized assessment of the epidemiological results, including exploration rate of increased PSA and interval cancer rate with stage.

## Notes

Supplement 1. eAppendix.

Supplement 2. Data Sharing Statement
