# Supplementary material for: Prediagnosis Prostate-Specific Antigen Testing History in Patients With Incident Prostate Cancer
Source: JAMA Netw Open. 2025 Nov 4;8(11):e2541321. doi: 10.1001/jamanetworkopen.2025.41321 (PMC12587197; doi:10.1001/jamanetworkopen.2025.41321)
Supplement: Supplement 1. — eAppendix. [file jamanetwopen-e2541321-s001.pdf]

Supplemental Online Content

Guittet L, Denis P, Tuppin P, et al. Prediagnosis PSA testing history in patients with incident prostate cancer. *JAMA Netw Open*. 2025;8(11):e2541321.
doi:10.1001/jamanetworkopen.2025.41321

eAppendix.

This supplemental material has been provided by the authors to give readers additional information about their work.

© 2025 Guittet L et al. *JAMA Network Open*.

Appendix.

1- Selection of incident prostate cancer (PCa) cases

Men with PCa between 2011 and 2022 who were alive in 2016 had: (a) the registration of PCa as a long-term disease (LTD), (b) a hospital stay in an acute-care setting with a ICD-10 code of PCa (D07.5;C61), (c) at least 3 reimbursements (2 if in large packs) of PCa medication in men aged ≥ 40 years, or (d) at least two prostate biopsies during a period of 4-24 months before any local PCa treatment (see below), considered as active surveillance of PCa.

The date of the first occurrence of any of these events was defined as the index date. PCa with an index date prior to 2016 were further excluded. Incident PCa cases in year n with no reimbursement for healthcare delivery during the year n-1, possibly out of France temporarily, were excluded due to uncertainty about the year of incidence.

For the exclusion of patients on androgen deprivation therapy (ADT) for a reason other than PCa, Patients who began ADT as a first-line treatment but had no LTD registration or hospital stays for PCa fulfilling at least one of the following criteria were excluded: (a) LTD registration for psychiatric or mental disorders, (b) ADT with leuprorelin, triptorelin or cyproterone, with no PSA test or procedures generating histological results indicating PCa in the six months before ADT initiation.

Patients with another cancer at the time of PCa occurrence were also excluded, to avoid misclassification of the stage at PCa diagnosis.

2- Stage definition

Stage at diagnosis is not recorded in the SNDS. It was therefore established according to the date of first ICD-10 code for metastasis, if any, and first-line treatment sequences based on medical guidelines.

Patients with: (a) a metastasis ICD-10 code (C77.- ;C78.-;C79.-), (b) systemic chemotherapy, (c) long duration of androgen deprivation therapy (ADT) (≥6 months) or new hormonal treatments other than darolutamide, (d) medical interventions targeting metastases, or (e) surgical castration initiated within six months of index date were considered as late-stage PCa.

Patients with: (a) local treatment (radical prostatectomy, high-intensity focused ultrasound, brachytherapy or prostate radiotherapy), (b) a short course of ADT (<6 months), or (c) active surveillance defined as repeated biopsies within a period of 4 to 12 months before any treatment as first-line treatment were considered as localized PCa.

The remaining patients were considered to have unstaged PCa.

© 2025 Guittet L et al. *JAMA Network Open*.
