# Supplementary material for: Prediagnosis Prostate-Specific Antigen Testing History in Patients With Incident Prostate Cancer
Source: JAMA Netw Open. 2025 Nov 4;8(11):e2541321. doi: 10.1001/jamanetworkopen.2025.41321 (PMC12587197; doi:10.1001/jamanetworkopen.2025.41321)
Supplement: Supplement 2. — Data Sharing Statement [file jamanetwopen-e2541321-s002.pdf]

Data Sharing Statement

Guittet. Prediagnosis Prostate-Specific Antigen Testing History in Patients With Incident Prostate Cancer. *JAMA Netw Open*. Published November 04, 2025. doi:10.1001/jamanetworkopen.2025.41321

Data
Data available: No
